# COVID-19 and SARS-CoV-2 Variants: Current Challenges and Health Concern

**DOI:** 10.3389/fgene.2021.693916

**Published:** 2021-06-15

**Authors:** Md. Zeyaullah, Abdullah M. AlShahrani, Khursheed Muzammil, Irfan Ahmad, Shane Alam, Wajihul Hasan Khan, Razi Ahmad

**Affiliations:** ^1^Department of Basic Medical Science, College of Applied Medical Sciences, King Khalid University (KKU), Abha, Saudi Arabia; ^2^Department of Public Health, College of Applied Medical Sciences, King Khalid University (KKU), Abha, Saudi Arabia; ^3^Genomic Science Academy, Muzaffarpur, India; ^4^Department of Medical Laboratory Technology, College of Applied Medical Sciences, Jazan University (JU), Jizan, Saudi Arabia; ^5^Department of Chemical Engineering, Indian Institute of Technology Delhi, New Delhi, India; ^6^Department of Chemistry, Indian Institute of Technology Delhi, New Delhi, India

**Keywords:** COVID-19, SARS-CoV-2 variant, therapeutics, vaccine efficacy, wave of infection

## Abstract

The ongoing coronavirus disease 2019 (COVID-19) outbreak in Wuhan, China, was triggered and unfolded quickly throughout the globe by severe acute respiratory syndrome coronavirus 2 (SARS-CoV-2). The new virus, transmitted primarily through inhalation or contact with infected droplets, seems very contagious and pathogenic, with an incubation period varying from 2 to 14 days. The epidemic is an ongoing public health problem that challenges the present global health system. A worldwide social and economic stress has been observed. The transitional source of origin and its transport to humans is unknown, but speedy human transportation has been accepted extensively. The typical clinical symptoms of COVID-19 are almost like colds. With case fatality rates varying from 2 to 3 percent, a small number of patients may experience serious health problems or even die. To date, there is a limited number of antiviral agents or vaccines for the treatment of COVID-19. The occurrence and pathogenicity of COVID-19 infection are outlined and comparatively analyzed, given the outbreak’s urgency. The recent developments in diagnostics, treatment, and marketed vaccine are discussed to deal with this viral outbreak. Now the scientist is concerned about the appearance of several variants over the globe and the efficacy of the vaccine against these variants. There is a need for consistent monitoring of the virus epidemiology and surveillance of the ongoing variant and related disease severity.

## Introduction

As of 10th April 2021, more than 134,308,070 cases with coronavirus disease 2019 (COVID-19) were diagnosed including 2,907,944 deaths (WHO, 2021b). In December 2019, COVID-19 was first announced in Wuhan, the Hubei province and a major transportation center in China, where several pneumonia cases linked to a recently discovered β-coronavirus. The WHO and the International Committee’s Coronavirus Study Group (CSG) proposed the newest coronavirus as SARS-CoV-2, declared on 11th February 2020. It spreads rapidly to other regions within the nation and, after a month, to other nations worldwide, affecting over 210 countries and territories. The World Health Organization (WHO) announced in January 2020 that COVID-19 is a worldwide emergency, and the organization later declared its occurrence to be a pandemic in March 2020 (Ahmed et al., 2020).

There have been two other epidemics of coronaviruses (CoVs) in the last 20 years. A widespread outbreak, starting in China, has aggravated SARS-CoV which fascinated two dozen countries with around 8,000 cases and 800 fatalities. Middle East respiratory syndrome coronavirus (MERS-CoV), which started in Saudi Arabia, has about 2,500 cases and 800 fatalities and continues to cause stray cases (Cascella et al., 2021). Acute respiratory distress syndrome (ARDS), such as alternative diseases causing respiratory disease/pneumonia, like Middle East Respiratory Syndrome (MERS) and Severe Acute Respiratory Syndrome (SARS), may also be caused by COVID-19 (Gibson et al., 2020). The basic reproduction number (R0) of SARS-CoV-2 in some studies is estimated to be approximately 2.2 (Riou and Althaus, 2020), or even higher (range from 1.4 to 6.5) (Liu Y. et al., 2020), and family clusters of pneumonia outbreaks indicate that the COVID-19 epidemic is growing increasingly through human-to-human transmission (Chan et al., 2020b). This review paper summarizes the findings on the epidemiology, clinical presentations, diagnosis, treatment, vaccine efficacy against several variants of SARS-CoV-2, and the wave of infection of COVID-19.

## Coronaviruses Characteristic, Genome Organization and Transmission

### History of Coronaviruses

Coronaviruses (CoVs) became the main pathogens of rising respiratory disease outbreaks. CoVs are the members of the Coronaviridae family within the order Nidovirales. Corona represents crown-like spikes on the outer surface of the virus, hence it is called a coronavirus (Richman et al., 2020). Coronaviruses are positive-sense enveloped RNA viruses varying in diameter from 60 to 140 nm (Zhu H. et al., 2020). The virus contains a single-stranded RNA, starting from 26 to 32 kbs in length, as a nucleic acid material. CoVs have four distinct genera: (i) α-coronavirus (alphaCoV), (ii) β-coronavirus (betaCoV), possibly present in bats and rodents, while avian species are presumably characterized by (iii) δ-coronavirus (deltaCoV), and (iv) γ-coronavirus (gammaCoV) (Perlman and Netland, 2009). Severe acute respiratory syndrome coronavirus (SARS-CoV), H1N1 2009, H5N1 influenza A and MERS-CoV cause acute lung injury (ALI) and ARDS, which brings on pulmonary failure and lead to fatality (MERS-CoV). These viruses were believed to only affect animals until an outbreak of severe acute respiratory syndrome (SARS) caused by SARS-CoV began in the Chinese province of Guangdong in 2002 (Zhong et al., 2003). A decade later, in Middle Eastern countries, another pathogenic coronavirus, known as the MERS-CoV, became prevalent (Wang et al., 2013).

At the end of 2019 recently, Wuhan, a growing business hub in China, experienced the outbreak of a unique coronavirus (a member of the β coronavirus cluster) that killed over 1,800 and infected over 70,000 people the initial fifty days of the epidemic. Chinese researchers named the unique virus as the Wuhan coronavirus or 2019 novel coronavirus (2019-nCov). The International Committee on Taxonomy of Viruses (ICTV) has designated the virus as SARS-CoV-2 and the disease as coronavirus disease 2019 stated as COVID-19 (Cui et al., 2019). In history, SARS-CoV (2003) infected 8,098 people with a 9 percent mortality rate in 26 countries worldwide, on the other hand, novel coronavirus (2019) infected more than 130,459,184 people with a 2.7 percent mortality rate in more than 215 countries till date. Continuous monitoring of SARS-CoV-2 by humans or animals is crucial for disease control because of the unstable nature of RNA.

### Characteristics of COVID-19, Origin and Risk Factors

SARS-CoV-2 contains positive single stranded RNA as genetic material. Electron microscopic study showed that the virus has a traditional crown-like structure due to glycoprotein spikes existence on its envelope (Perlman and Netland, 2009). Age may be a robust risk issue for a severe sickness, complications, and death (Guan et al., 2020). In patients from China with no reported underlying medical problems, there was an overall case fatality of 0.9 percent. In patients with comorbidity, case fatality was found to be greater: 10.5% in patients with cardiovascular disease, 7.3% in patients with diabetes, and around 6% in patients with chronic respiratory disease or cancer (Guan et al., 2020; Abdelhafiz et al., 2021; Bae et al., 2021; ElAbd et al., 2021). It has been also associated with depressive and Anxiety Symptoms of Healthcare Workers (Peng et al., 2021). Increased illness severity and adverse effects have all been associated with prior stroke, diabetes, chronic lung and kidney disease. People with severe heart disease, including heart failure, congenital heart disease, coronary artery disease, cardiomyopathy and hypertension of the lungs, are at greater risk of COVID-19 severe disease.

### Genome Structure and Organization

**FIGURE 1:**
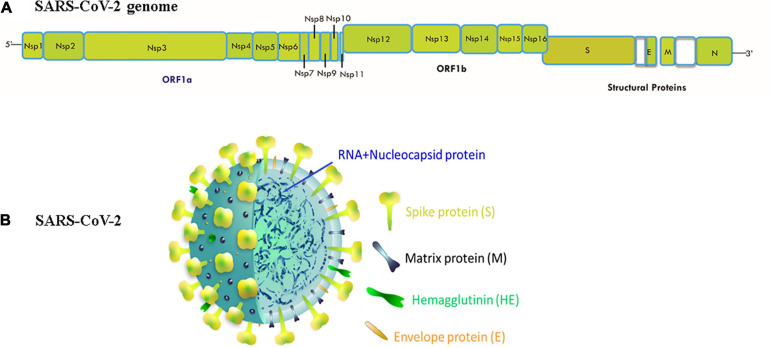
**(A)** Genomic organization of SARS-CoV-2: 5′ and 3′ terminal sequences of the genome of SARS-CoV-2. 5′-replicase ORF1ab-spike (S)-envelope (E)-matrix (M)-nucleocapsid (N)-3′ is the gene order. At the expected position shown in the figure, Nsp1, Nsp2, Nsp3, Nsp4, Nsp5, Nsp6, Nsp7, Nsp8, Nsp9, Nsp10, Nsp11, Nsp12, Nsp13, Nsp14, Nsp15, Nsp16, S, E, M, N serve as various ORF genes in the frame. **(B)** Structure of SARS CoV-2:The genomic size of β-coronavirus ranges from 29 to 32 Kb. The virion has a genomic RNA and phosphorylated nucleocapsid (N) protein with a nucleocapsid that is concealed within phospholipid bilayers and coated with the spike glycoprotein trimmer (S). In the envelope of the virus, the matrix (M) protein hemagglutinin-esterase (HE) and envelope (E) protein are placed amid S proteins.

In the past twenty years, SARS-CoV-2 is the third speedily emerged zoonotic RNA coronavirus, which evolved in the human population (Munster et al., 2020). SARS-CoV-2 was first isolated from Wuhan Jinyintan Hospital on December 30, 2019, in the bronchoalveolar lavage fluid (BALF) of three COVID-19 patients (Zhu N. et al., 2020). On December 31, 2019, delegates from the Chinese Center for Disease Control and Prevention (CDC) moved to Wuhan for field investigations, and on January 6, 2020, a sample of a new virus was isolated and confirmed as a pathogen of unidentified pneumonia. In the following days, the virus’s genome-wide sequence was decoded. China announced the genetic sequence of the novel coronavirus on January 12, 2020 for countries to use in designing specialized diagnostic kits (Huang C. et al., 2020; WHO, 2020; Zhu H. et al., 2020). The CoVs are classified according to the genotype and serology into four separate subfamilies: α, β, γ, and δ-CoVs. The α- and β-CoVs cause human CoV infections (Weiss and Leibowitz, 2011). The β-CoVs (enveloped, non-segmented, positive-sense single-stranded RNA) includes SARS coronavirus (SARS-CoV) and the MERS coronavirus (MERS-CoV) (Weiss and Leibowitz, 2011). The genome organization of SARS-CoV-2 and their structure has shown in Figure 1. The size of virus genome ranging from 29.9 kb. These viruses may cause diseases of the respiratory, enteric, hepatic, and neurological forms (Weiss and Leibowitz, 2011; De Wilde et al., 2018). Genome-based phylogenetic investigation expresses that SARS-CoV-2 shares 79.5 percent and 50 percent sequence homology to SARS-CoV and MERS-CoV, respectively (Lu et al., 2020; Zhou et al., 2020; Zhu N. et al., 2020). However, between the seven preserved replicase domains in ORF1ab of SARS-CoV-2 and SARS-CoV (Zhou et al., 2020), there is 94.6 percent sequence homology and less than 90 percent sequence homology between those of SARS-CoV-2 and other β-CoVs (Zhu N. et al., 2020), entailing that SARS-CoV-2 resides in lineage B (Sarbecovirus) of β-CoVs (Wu et al., 2020). SARS-CoV-2 has a nucleocapsid consisting of a nucleocapsid (N) protein and genomic RNA. In phospholipid bilayers, the nucleocapsid is buried and coated with two separate forms of spike proteins: some CoVs only share the spike glycoprotein trimmer (S), which totally exists in CoVs, and hence the hemagglutinin-esterase (HE). The membrane (M) and the envelope (E) proteins are situated among the S proteins inside the envelope of the virus (Li G. et al., 2020; Wu et al., 2020).

### Epidemiology

Based on the data generated by the WHO coronavirus disease (COVID-19) dashboard, 130,459,184 confirmed COVID-19 cases had been reported, including 2,842,325 deaths. Of note, 11 percent cases are fatal (as of 6th April 2021 by WHO weekly epidemiological update on COVID-19). There have been incidents in more than 215 countries to date. There are 56,589,190 confirmed cases in the Americas; 45,877,941 in Europe; 15,212,235 in South-East Asia; 7,693,094 in the Eastern Mediterranean; 7,693,094 in Africa; and 1,965,683 in the Western Pacific. The highest fatal cases have been recorded in the Americas (1,368,633) followed by Europe (980,586), and South-East Asia (222, 054) (WHO, 2021a).

## Diagnostic Methods

COVID-19 must be diagnosed immediately to begin proper therapy, avoid further viral transmission, and effectively eliminate the virus from circulation. Table 1 summarized the method for detecting novel coronavirus with accuracy and rapid diagnosis. Molecular methods are the first-line techniques for the certification of reported cases of novel coronaviruses. Nucleic acid monitoring is a powerful laboratory diagnostic tool. Other methods include viral antigen or serological antibody testing, which are useful in identifying new coronavirus infections (Chen et al., 2015). Like other emerging viruses, antibody and viral antigen detection methods are undertaken after the viral genome’s identity. Following the start of the Wuhan, China outbreak on January 10, 2020 (WuhanHu-1, GenBank Accession No. MN908947), SARS-CoV-2 genomic sequence available in databases, which aided the advancement of standardized COVID-19 laboratory PCR protocols in January 2020 (Debing et al., 2014).

**TABLE 1:** Different diagnostics methods for COVID-19.

**Method**	**Characteristics/features**	**Limitations/disadvantages**	**References**
Antigen-detection	Low complexity; rapid; easy to perform	Best used to identify acute or early infection; more prone to false negatives	Chen et al., 2016; Diao et al., 2020; Sheridan, 2020
Antibody-detection	Fast, robust and easy to perform; requiring only a small amount of sample	Unable to detect the presence of infection during the early stage of disease; cross-reactivity	Li Z. et al., 2020; Long Q.X. et al., 2020; Sheridan, 2020; Zhang et al., 2020; Zhao et al., 2020a
RT-qPCR	Specific, sensitive and simple quantitative assay, which greatly helps in the diagnosis of early infection	Costly and time consuming to perform; more prone to false negatives or low value	Chan et al., 2020a; Corman et al., 2020; Lan et al., 2020; Wang Y. et al., 2020
CT Scan	Available earlier; check severity of condition; check possible infection	Expensive; unable to distinguish from other viral pneumonias; hysteresis of abnormal CT imaging	Bernheim et al., 2020; Huang Y. et al., 2020; Li L. et al., 2020; Liu F. et al., 2020; Zhao et al., 2020c
CRISPR-based Detection	High sensitivity and specificity with efficiency and no requirement for elaborate instrumentation	Certain biological safety hazards brought by the retention and operation of patient samples	Gootenberg et al., 2018; Abbott et al., 2020; Broughton et al., 2020; Guo et al., 2020

In patients with acute respiratory infections during international health emergencies, the real-time RT-PCR test is a delicate and valuable tool to identify respiratory pathogens (Wang C. et al., 2020). For the accelerated production of real-time RT-PCR diagnostic tests, the genome sequence was used to construct special primers and probes to detect SARS-CoV-2 (Corman et al., 2020). Another useful and exciting method involves clustered often interspaced short palindromic repeats (CRISPR) technology that is being expeditiously expanded in the molecular diagnostics landscape. The diagnostic assays based on CRISPR benefit expeditiously from high sensitivity and specificity. Many clinicians have recommended that a major accessory diagnostic technique be computed tomography (CT) scans (Yang and Yan, 2020; Zhao et al., 2020c). Based on host antibody detection, one sort of fast diagnostic assay has become accessible more recently (Sheridan, 2020). Another form of the rapid diagnostic assay (RDT) that detects the presence in a respiratory tract sample of viral antigens revealed by the SARS-CoV-2 virus is low in complexity. It will usually offer results within half an hour (Sheridan, 2020). If the virus is actively replicating, the antigen(s) identified are revealed; hence, such tests are best used to analyze acute or early infection. A minimum of five antigen-detection RDTs was understudy at this examination, although not all CE Markings are licensed or widely available.

## Potential Strategies to Treat COVID-19

### Potential Therapeutics

Few possible antiviral medications are being immediately prescribed to COVID-19 patients during the pandemic (Table 2). Remdesivir (GS-5734) may be an adenosine nucleotide 1’-cyano-substituted analog prodrug and demonstrated large-spectrum antiviral activity against so many RNA viruses (Ye et al., 2020; Matthias, 2021). Based on evidence from an *in vitro* cell line and a mouse model, Remdesivir can inhibit the NSP12 polymerase even when ExoN proofreading activity is perfect (Agostini et al., 2018). Remdesivir has been noted for successfully treating COVID-19 first US case (Holshue et al., 2020). Chloroquine is a repurposed medication for the treatment of COVID-19 with great promise. Chloroquine is often used to cure malaria for many years, a method that is not well understood for specific viral infections (Aguiar et al., 2018). Many mechanisms are being analyzed like disseminating SARS-CoV infection is inhibited by a potent concentration of chloroquine (Savarino et al., 2003; Vincent et al., 2005). Chloroquine has immunomodulatory properties and inhibits TNF-α and IL-6 synthesis and release.

**TABLE 2:** Different therapeutic agents used for COVID-19 treatment.

**Therapeutic agents**	**Proposed doses for COVID-19**	**Mechanism of action**	**Target diseases**
Remdesivir (GS-5734)	200 mg on day 1, then on days 2–10 100 mg	Nucleoside analog (terminates RNA synthesis) Interfering with virus post-entry	Ebola, SARS-CoV-2
Chloroquine (CQ)	500 mg each time, 2 times/day for 5–10 days (300 mg for chloroquine)	Increasing endosomal pH Autophagy inhibitors Inhibits viral RNA polymerase Immunomodulating Probably inhibit ACE2 cellular receptor	Antimalarial agent, autoimmune disease
Hydroxychloroquine (HCQ)		Hydroxychloroquine shares the same mechanism of action as chloroquine	SARS-CoV, MERS-CoV, SARS-CoV-2
Lopinavir and ritonavir	500 mg once, twice a day, for 2 weeks	Protease inhibitors inhibit coronavirus replication	HIV infection
Ribavirin	500 mg a day, 2–3 times daily, in conjunction with IFN-alpha or lopinavir/ritonavir	Nucleoside inhibitor (Interfering with the synthesis of viral mRNA)	Hepatitis C, SARS, MERS
Nelfinavir	400/100 mg (2 tablets of 200/50 mg) every 12 h	Protease Inhibitor	Solid Tumors, HIV
Umifenovir (Arbidol)	200 mg each time, 3 times/day	S protein/ACE2, membrane fusion inhibitor Inhibits the replication of coronavirus *in vitro*	Influenza infection
Favipiravir (T-705)	1,600 mg*2/first day followed by 600 mg*2/day	Nucleoside analog (RNA polymerase inhibitor)	Influenza A (H1N1), Ebola
Camostat mesilate (FoipanTM)	600 and 300 mg/day	Inhibits serine protease	SARS-CoV-2
Interferon-alpha (IFN-α)	5 million IU/ml, 2 times/day	Increase cellular immunity, Inhibits viral replication	Broad-spectrum antiviral
Tocilizumab	400 mg IV or 8 mg/kg × 1–2 doses Next dose 8–12 h after the first dose if insufficient response	Inhibits IL-6-mediated signaling (also reduce cytokine storm)	Rheumatoid arthritis
Dexamethasone	16 mg on days 1–5 and 8 mg on days 6–10	Inhibits inflammatory cells and suppress the expression of inflammatory mediators	MERS and SARS

**Most of these medicines should be used maximum up to 10 days. ACE2, angiotensin-converting enzyme 2; AST, aspartate aminotransferase; G6PD, glucose-6-phosphate dehydrogenase; HIV, human immunodeficiency viruses; IL-6, interleukin 6; IV, intravenous therapy; IU, international unit; URTI, upper respiratory tract infection; MERS, Middle East respiratory syndrome; SARS, severe acute respiratory syndrome.**

Furthermore, it is a new class of autophagy-inhibiting agents that inhibits viral infection and replication (Golden et al., 2015). Several experiments have shown that chloroquine has impacted glycosylation of SARS-CoV cellular receptors during infection as well as after infection in Vero-E6 cells (Vincent et al., 2005; Wang M. et al., 2020). The combination of remdesivir and chloroquine was shown to effectively inhibit SARS-CoV-2. Viral loads of β-coronavirus in COVID-19 patients have been reported to have decreased significantly in Korea following treatment with lopinavir/ritonavir (Lim et al., 2020). Ribavirin was primarily used in patients with or without concomitant steroid use during the outbreak of SARS in Hong Kong (Wenzel and Edmond, 2003; Morgenstern et al., 2005; Khamitov et al., 2008). IFNs of type 1 are antiviral cytokines contain several proteins to inhibit viral replication in targeted cells. Previous research found that IFN-β was more effective against SARS-CoV than IFN-α (Scagnolari et al., 2004). It has been widely suggested that convalescent plasma may be used to treat COVID-19 (Li H. et al., 2020). The monoclonal antibody (mAb) has been used to neutralize SARS-CoV and prevent the formation of syncytia between cells that express the S protein and those who reflect the ACE2 SARS-CoV receptor (Duan et al., 2005).

### Nanomedicine

Nanotechnology is a broad subject spanning many disciplines, such as materials science, physical science, biological science and medicine. The use of nanotechnology in medicine is attributed to as nanomedicines (Sardar et al., 2014; Tabish and Hamblin, 2020). Nanomaterials, nanoparticle, nanodrugs, nanoconstructs, nanotherapeutics, and nanocarriers are all terms used to describe nanomedicine (Ahmad et al., 2014a, 2015, 2021; Fornaguera and Garcia-Celma, 2017; Alam et al., 2018; Sadaf et al., 2019). Nanomaterials are used as carrier delivery vehicles for nanomedicines that allow these particles to travel to the desired target tissue and balance the concentration of the whole molecular concoction over time to ensure the precision and effectiveness. Nanoparticles have been widely used for decades due to their nanoscale size, mobility, multifunctionality, ability to adapt, increased solubility, personalized medications, early detection, and disease prevention (Ahmad and Sardar, 2015; Ahmad and Khare, 2018; Ghosh et al., 2018a,b; Soares et al., 2018). Nanomedicine has been used successfully to enhance treatment for a wide range of illnesses including neural, cancer, cardiopulmonary, and communicable diseases like HIV-1, HBV, influenza virus, and respiratory syncytial virus (Ahmad et al., 2014b; Ghosh et al., 2019, 2021a,b; Abd Ellah et al., 2020; Srivastava et al., 2021). Nanotechnology could play a potential role in diagnosis, treatment, and prevention of COVID-19 (Bhavana et al., 2020; Campos et al., 2020; Noori et al., 2020). Furthermore, numerous CoV-related patents have been enrolled in the area of nanotechnology (Abd Ellah et al., 2020). The global effect of the current pandemic is terrifying, and vaccine is the most effective option for preventing the transmission and combating novel CoV outbreaks. To design the vaccine, first describe the antigen, adjuvant, manufacturing method, and distribution system. The antigen is a pathogen-specific foreign material delivered to initiate the host and adjuvant immune response as a stimulatory agent to enhance the host immune response (Shin et al., 2020; Goel et al., 2021). Nanoparticles allow for multiple antigen presentations while also protecting antigens from degradation by the host cell system after administration and serving as carriers for target-based transmission. Two fundamental problems in vaccine design would resolve the efficient distribution of antigen to dendritic cells following dendritic cell activation to cause adaptive immunity (Reddy et al., 2007). The nanoparticles able to carry various antigens promote the activity of antigen-presenting cells, enhancing the immunogenicity and potency of administered vaccines through recognition by T cell receptors (Hashemzadeh et al., 2020). However, more research toward the use of nanoparticles in the development of promising CoVs vaccines is required (Abd Ellah et al., 2020).

### Therapeutic Vaccine

COVID-19 vaccines are being introduced across the world to develop immunity to the disease. Vaccines based on viral vectors, nucleic acid-based vaccines, attenuated vaccines, and protein-based vaccines have all contributed significantly to clinical trials. Today, 11 new vaccines are being carried out around the world. Among the companies are Pfizer/BioNtech, Moderna, Oxford/AstraZeneca, Bharat Biotech, Sputnik V, SinoVec, Sinopharm, CanSino, Johnson and Johnson, Novavex and EpiVecCorona. Aside from these, 183 vaccines are currently in preclinical production, with 97 in clinical trials. Some current marketed vaccines are listed in Table 3. Researchers must ensure that the COVID-19 vaccine is safe for the aged and people with comorbidities (such as Heart failure, Post cardiac transplant, Heart Disease, stroke, and Hypertension/Diabetes on treatment, Kidney/Liver/Hematopoietic stem cell transplant, Severe respiratory disease), as these groups of people are the most vulnerable (Haynes et al., 2020; Jeyanathan et al., 2020; Zhao et al., 2020b). The vaccine’s level of safety must also be determined, and patients may need more than one dose to maintain viral immunity. Several vaccines and their efficiency against variants of the virus that causes COVID-19 have been shown in Table 4.

**TABLE 3:** List of COVID-19 marketed vaccines: manufacturers, trade names, platforms and existing status of approval for usage in various countries are mentioned in the representative table.

**Inventor**	**Name of vaccine**	**Platform**	**Status for emergency use**
Moderna	mRNA-1273	mRNA encapsulated in lipid nanoparticle	Approved in Switzerland. Emergency use in U.S., U.K., E.U., others.
Bharat Biotech	Covaxin, BBV152 A, B, C	Whole virion Inactivated SARS-CoV-2 vaccine + adjuvant	Emergency use in India.
Pfizer/BioNtech	Comirnaty, tozinameran, BNT162b2	mRNA encapsulated in lipid nanoparticle	Emergency use in U.S., E.U. etc., Approved in several countries.
OXFORD AstraZeneca	AZD1222 (Covishield in India)	Attenuated adenoviral vector (non-replicating) from chimpanzee ChAd	Emergency use in U.K., E.U., India, and other countries.
Sinovac	CoronaVac, PiCoVacc	Inactivated coronavirus- Done using chemical beta-propiolactone	Approved in China, Emergency use in Brazil, Singapore, Malaysia, and Philippines
Gamaleya	Sputnik V, Gam-COVID-Vac	Viral 2 vector-based vaccine- rAd26 vector and rAd5 vector	Early use in Russia, Emergency use in other countries.
Sinopharm	BBIBP-CorV	Inactivated SARS-CoV-2 vaccine (Vero cell)	Approved in China, U.A.E., Bahrain, Emergency use in Egypt, other countries.
CanSino	Convidecia, Ad5-nCoV	Adenovirus based viral vector (Ad5)- Non-Replicating	Emergency use in China and Mexico
Johnson and Johnson	Ad26.COV2.S	Adenovirus based viral vector (Ad26)- Non-Replicating	Applied for emergency use authorization in United States
Vector Institute	EpiVacCorona	Chemically synthesized peptide antigens of SARS-CoV-2 proteins	Early use in Russia.
Novavax	NVX-CoV2373	S Protein adjuvanted with recombinant novavax protein	Early use in United Kingdom and Australia

**TABLE 4:** Description of SARS-CoV-2 vaccine trial efficacy and viral neutralization of B.1.1.7, 501Y.V2, and P.1 variants vs. preexisting variants (Abdool Karim and de Oliveira, 2021).

**Vaccine**		**Neutralization by pseudovirion/Live viral plaque assay**			
	**Preexisting variants with efficacy in preventing clinical/severe COVID-19 (%)**	**B.1.1.7 variant**	**501Y.V2 variant**	**P.1 variant**	**References**
BNT162b2 (Pfizer)	95/90	Decrease by 2×	Decrease by ≤ 6.5×	Decrease by 6.7×	Polack et al., 2020; Garcia-Beltran et al., 2021; Wang et al., 2021
mRNA-1273 (Moderna)	94/100	Decrease by 1.8×	Decrease by ≤ 8.6×	Decrease by 4.5×	Baden et al., 2021; Garcia-Beltran et al., 2021; Shen et al., 2021; Wang et al., 2021; Wu et al., 2021
NVX-CoV2373 (Novavax)	89/100	Decrease by 1.8×	NA	NA	Shen et al., 2021
Sputnik V (Gamaleya)	92/100	NA	NA	NA	Logunov et al., 2021
AZD1222 (AstraZeneca)	67/100	NA	Decrease by ≤ 8.6× to complete immune escape	NA	Madhi et al., 2021; Voysey et al., 2021
BBIBP-CorV (Sinopharm)	79/NA	NA	Decrease by 1.6×	NA	Huang et al., 2021

## Variants of SARS-CoV-2 and Its Correalation With Infection

**FIGURE 2:**
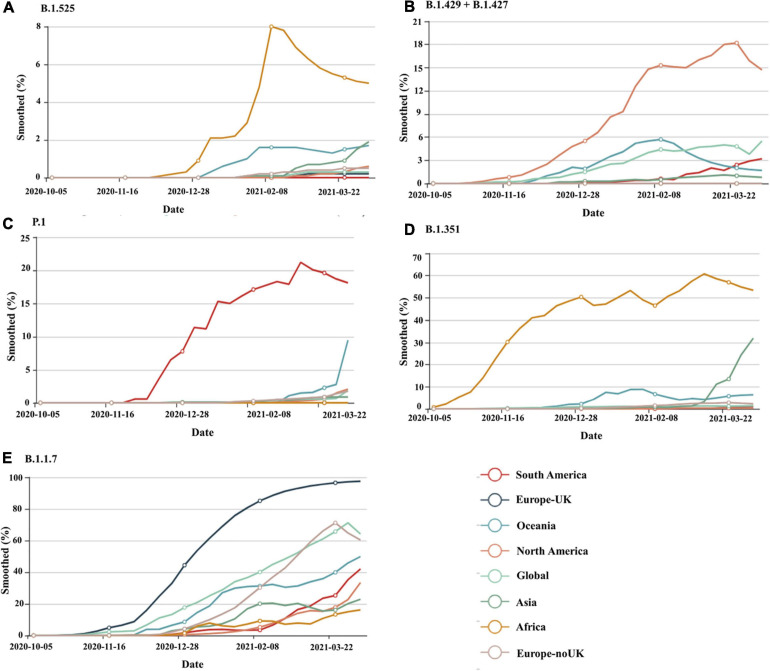
The worldwide distribution of circulating SARS-CoV-2 lineages, **(A)** B.1.1.7, **(B)** B.1.351, **(C)** P.1, **(D)** B.1.429 + B.1.427, **(E)** B.1.525 on a time scale for South America, Europe United Kingdom, Oceania, North America, Global, Asia, Africa, and Europe-noUK. Source: https://www.gisaid.org/hcov19-variants/for the most recent global SARS-CoV-2 variant as of April 2021.

Viruses continuously evolve by replication, and new forms of a virus are predicted to develop over time (Yan et al., 2021). Throughout the pandemic, several strains of SARS-CoV-2 have been identified in the United States and around the world. SARS-CoV-2 variants are classified into three categories by a US government interagency group as variant of Interest, variant of concern, and variant of high consequence. The B.1.1.7, B.1.351, P.1, B.1.427, and B.1.429 versions are listed as variants of concern. Variant of concern showing a genetic variation associated with enhanced transmissibility, more serious illness (e.g., increased hospitalizations or deaths), substantial reduction of neutralization by antibodies produced during previous infection or vaccination, decreased efficacy of medications or vaccinations, or diagnostic identification failures for this variant. These having more impact on diagnostics, treatments, or vaccines. Also, there is evidence of reduced neutralization by antibodies generated during a prior infection or vaccine (CDC, 2021a). B.1.1.7, B.1.351, P.1, B.1.429 + B.1.427, and B.1.525 are the five genetic variants of concern of SARS-CoV-2 that have been detected thus far from various geographical locations around the world and their time-dependent appearances are depicted in Figure 2^1^. The different lineage characteristics including origin, mutation in the spike protein and attributes are mentioned in Table 5. RNA viruses, such as SARS-CoV-2, were more susceptible to genetic variation than DNA viruses, resulting in a variety of variants (Khan et al., 2015; Raghuram et al., 2015; Haider et al., 2018). The variants may appear to propagate even more rapidly than the others, contributing to more cases of COVID-19 (Toyoshima et al., 2020). At the starting of 2020, a SARS-CoV-2 mutant with a D614G mutation in the gene encoding the spike protein appeared. The D614G mutation gradually replaced the original SARS-CoV-2 strain discovered in China and had become the dominant type of the virus circulating worldwide. Studies in human respiratory cells and animal models showed that the virus strain with the D614G substitution has higher infectivity and dissemination than the original virus strain. The D614G replacement in the SARS-CoV-2 virus has no impact on the efficacy of current laboratory diagnostics, therapeutics, vaccinations, or public health prevention initiatives (Hou et al., 2020; Plante et al., 2021). A rise in the number of cases would make more demand on health care services, infrastructure and generate more fatalities within our society. Current research indicates that antibodies produced by vaccines administered to Indigenous people respond to these variants (Jeyanathan et al., 2020; Abdool Karim and de Oliveira, 2021). This is also being studied, and new experiments are on the way for better understanding.

**TABLE 5:** Major SARS-CoV-2 lineage: research describes how often a vaccine will protect people affected by these strains.

**lineage**	**Country**	**Spike protein substitution**	**Disease severity**	**References**
B.1.1.7	United Kingdom	N501Y, A570D, D614G, P681H, T716I, S982A, D1118H, 69/70/144 deletion	Increased transmission, severity of hospitalization based on case fatal rates	Arif, 2021; CDC, 2021b; Galloway et al., 2021; Ramírez et al., 2021
501Y.V2 or B.1.351	South Africa	Shares some mutations with B.1.1.7. K417N, E484K, D214G, A701V 241/242/243 deletion	There is no indication that this variant has any impact on disease incidence.	CDC, 2021b; Faria et al., 2021; Wibmer et al., 2021
501Y.V3 or P.1	Brazil	17 unique mutations, including three in the receptor binding domain of the spike protein K417T, E484K, and N501Y	The advent of this mutation raises questions about a rise in transmissibility or a proclivity for SARS-CoV-2 re-infection in individuals.	CDC, 2021b; Faria et al., 2021
B.1.427 + B.1.429	United States	D614G, L452R S13I, W152C	Around 20% increased transmissibility, reduced neutralization by convalescent and post vaccination sera	Deng et al., 2021
VUI-21FEB-03 or B.1.525	U.K, NIGERIA	A67V, E484K, D614G, Q677H, F888L, 69/70/144 deletion	Reduced neutralization by convalescent and post vaccination sera	Jangra et al., 2021

**The table shows the main variants, countries of origin, new mutations found, and the risks they pose to public health.**

## Waves of SARS CoV-2

The second and third waves of COVID-19 are causing anxiety in populations worldwide (Kumar et al., 2020; Van Damme et al., 2020). The mutation in the coronavirus’ genetic code is the well-known source of multiple waves. The virus get more time to mutate and find ways to evade or deceive antibodies if vaccination is done slowly. Experts predict a third COVID-19 wave before the country has a chance to heal. In the United States, three concurrent epidemiological waves of COVID-19 dissemination have been reported, each with three distinct structural types. The first of three waves was catalyzed by early propagation in the United States. On January 26, 2020, the first recorded COVID-19 event in the United States happened in Washington State. By the end of February, the novel coronavirus had dispersed quickly through the continental United States, infecting a variety of environments such as long-term care hospitals, assisted-living facilities, and nursing homes. By mid-march, every state in the United States had recorded incidents. The second COVID-19 wave was presented to all U.S. states by mid-march of 2020. The key cause of the outbreak was long-term population dissemination in densely populated communities and towns. The third COVID-19 wave was presented to all U.S. states after April 2020. The key point of the outbreak was prisons/jails and Immigration and Customs Enforcement (ICE) detention facilities (Long S.W. et al., 2020; Solis et al., 2020). People are becoming tired of the constant restrictions on their travel and other disruptions in their lives and the economic effects of COVID-19, which is exacerbating the situation. However, the most alarming aspect is what we see in terms of demographics and COVID-related symptoms, rather than the rising number of cases and hospitalizations. The spike protein of almost all strains in the second wave has a D614G amino acid substitution, a polymorphism related to improved dissemination and infectivity. The scientific community must be alert and ready to learn about the causes, nature, and mechanism of potential waves of pathogens and the impact on SARS CoV-2 growth and any associated host immune response and therapeutic strategies (Seitz et al., 2020).

## Conclusion and Future Perspectives

The epidemic of COVID-19 swept quickly through China and expanded to more than 216 countries/territories/areas outside of China. Since the sudden emergence and fast spread of COVID-19 threaten public health and the economy, it is important to advance strategies to accommodate the virus’s spread. Identifying the novel coronavirus is progressing, and scientists are concentrating their efforts on developing antiviral therapies and vaccines. This novel virus epidemic has posed a threat to the global economic, medical, and public health system, and only time can tell how the virus’s global spread can affect our everyday lives. Furthermore, future outbreaks related to viruses and pathogens of zoonotic origin are expected to endure. In addition to curbing this outbreak by primary healthcare providers, efforts should also be made to prepare systematic steps to avoid potential zoonotic outbreaks. Care is mostly supportive; the function of antiviral agents has not yet been determined. In hospitals with contact and droplet precautions, prevention includes home isolation of suspected cases and people with mild diseases and stringent infection control measures. The global effect of this latest crisis on public health, social well-being, and economic prosperity is still unknown. Vaccination is needed to combat the COVID-19 virus, and vaccine development is contingent on further research into variant strains.

## Author Contributions

MZ, AA, KM, IA, SA, WK, and RA conceptualized, prepared, and critically reviewed the manuscript. All authors contributed to the article and approved the submitted version.

## Conflict of Interest

The authors declare that the research was conducted in the absence of any commercial or financial relationships that could be construed as a potential conflict of interest.

## Footnotes

1 https://www.gisaid.org/hcov19-variants/
